# Grand Challenges in Food Allergy

**DOI:** 10.3389/falgy.2021.668479

**Published:** 2021-03-10

**Authors:** Ronald van Ree

**Affiliations:** Departments of Experimental Immunology and of Otorhinolaryngology, Amsterdam University Medical Center, Location AMC, Amsterdam, Netherlands

**Keywords:** food allergy, mechanisms of sensitization, diagnosis, intervention, immunotherapy, prevention

## Food Allergy: a Growing Public Health Problem

Popularly, adverse reactions to food, ranging from anaphylaxis and gluten hypersensitivity to lactose intolerance and even food poisoning, are often shoved together under the term “food allergy.” To avoid confusion, here we restrict food allergy to adverse reactions with an immunological basis, i.e., to inflammatory diseases of the immune system. These include IgE antibody-driven immediate-type reactions to food, but also non-IgE mediated immune disorders, such as celiac disease, eosinophilic esophagitis, and food-protein-induced enterocolitis. Food allergy can affect many different organ systems, such as the skin, the gastro-intestinal tract, and the respiratory tract. Especially when affecting the cardiac and/or neurological system, reactions can be life-threatening (anaphylactic shock). In high-income countries, the prevalence of allergies, including those to food (1), has increased during recent decades, a trend that is now also spreading to upcoming economies (2). Changes in environment, increased urbanization, global warming, decreased exposure to early-life infections, and changes in lifestyle and diet all play their part in this development. Food allergy and eczema are usually the earliest expressions of allergic disease, starting at very young age, often in the first year of life. It is considered the start of the so-called allergic march, in which the allergic phenotype with time spreads toward respiratory symptoms, such as allergic asthma and allergic rhinitis, induced by exposure to indoor (house dust mites, pets, molds, and cockroach) and outdoor allergens (mainly pollen) (3). In particular pollen allergies subsequently lead to further increase in food allergies based on IgE cross-reactivity (4). Food allergy can severely affect the quality of life of patients and of their caretakers. Until recently, the only treatment was avoidance, and in case of accidental exposure, rescue medication. Recent scientific advancements have opened exciting new avenues for diagnosis, prevention, management, and treatment. What are the current grand challenges for food allergy research and management?

## Food Allergy: How Does It Start and How Can We Prevent It?

The classical paradigm of sensitization to food occurring orally, has shifted in favor of alternative routes, such as the skin and possibly the airways (5). Several epidemiological observations have put the skin in the center of attention. First, there was the observation that the use of ointments containing peanut oil increased the risk of peanut allergy (6). Then, food allergy was found to be associated with SNPs in the skin barrier protein filaggrin that lead to impairment of barrier function (7). This was followed by reports that the presence of peanut allergen in house dust samples was associated with the development of peanut allergy, but only in those patients that carry the loss-of-function SNPs for filaggrin (8). Altogether, sensitization via the skin likely plays an important role in the development of food allergy. It can however not be excluded that sensitization also occurs via the respiratory tract; there is some evidence that filaggrin mutations promote allergic disease in the airways as well (9). Whether the oral route is involved in sensitization to food at all is a matter of debate, but obviously the default programming for the oral route is more toward tolerance, to be compatible with life. For designing effective strategies to prevent food allergy, better understanding of the relative importance of the various possible routes of sensitization remains of great importance.

For long time, the advice to atopic parents has been to avoid exposure of their offspring to house dust mites and pets as much as possible. The outcome of this proved to be less straightforward or sometimes even counter-productive (10, 11). For children at risk of developing food allergy, e.g., when they develop eczema in the first months of life, the thought has long been to delay introduction of solid foods, in particular of those known to be highly allergenic, such as peanut. The first cracks in the concept of delayed introduction came from studies comparing Jewish communities in Israel and London (12). Early-life high exposure to peanut in Israel proved to result in low prevalence of peanut allergy, compared to London, where delayed introduction was common practice. This led to the seminal LEAP intervention study, demonstrating that early introduction of peanut protected against the development of peanut allergy in an at-risk population (13). Other studies have followed, mainly for peanut and egg, some confirming protection, but other not being successful (14). Nevertheless, from being seen as a risk factor, early-life oral exposure is now becoming a promising avenue for primary (and secondary?) prevention, but the reported contradictory results stress the need for further studies. Why does it work in one and not in another population? Why is it successful for one food and not for a different one? How high and how frequent should exposure be? Which new-borns to select for early introduction?

Early-life exposure to food like, e.g., peanut never happens in isolation. Together, environmental, (epi)genetic, dietary, and life-style factors influence the way the immune response to food is skewed. It is now well-recognized that the microbiome plays an important role in shaping immune responses (15). Route of sensitization to food will determine which microbiome is involved, i.e., of the intestines, the skin or even the airways. Microbiomes are shaped by route of delivery of new-borns, by diet (e.g., fresh vs. processed, unpasteurized vs. pasteurized, according to season or not), environmental exposure to pathogenic and non-pathogenic microbes and parasites, viral infections, vaccinations and antibiotics use. Many of these co-factors present quite differently in affluent countries, upcoming economies and low-income countries (16). This socio-economic and cultural gradient offers great opportunities to study the role of all these factors in the development of food allergy, and to search for effective preventive and therapeutic strategies.

## Food Allergy: How to Diagnose It?

The gold standard for food allergy diagnosis is an oral food challenge (OFC), preferably double-blind placebo-controlled (DBPCFC). In recent years, component-resolved diagnostics (CRD) have been shown to significantly improve the predictive value of *in vitro* tests, thereby reducing the need for OFCs (17). In particular 2S albumins from legumes (e.g., Ara h 2) and tree nuts (e.g., Cor a 14) have proven to be useful diagnostic tools. A matter of debate remains what the position of microarray-like CRD formats is in the diagnostic work-up of subjects with a suspicion of food allergy (18). They can provide a very comprehensive sensitization profile with very little serum, but sensitivity is low compared to singleplex CRD. Moreover, one may argue that they provide much information not asked for, perhaps sometimes creating more confusion than providing clarity in daily clinical practice.

In parallel to the advent of CRD in food allergy diagnosis, *in vitro* tests for biological activity of food allergen-specific IgE, such as the basophil activation test (BAT), have demonstrated good diagnostic performance as well (19). It is likely that a “marriage” of CRD and BAT, e.g., BAT with purified Ara h 2, may further increase diagnostic accuracy. Availability of facilities to perform BAT in non-academic daily clinical practice is a potential hurdle toward broad implementation, and therefore the development of molecular skin prick tests may offer a solution here.

Molecular diagnostics can also help to better distinguish different disease phenotypes and endotypes, in particular to estimate risks of severe reactions. In some studies, convincing evidence has been presented that IgE against, e.g., Ara h 2 (20) or Cor a 14 (21) is associated with more severe reactions, but this was not found in all studies. It will be important to elucidate which demographic and clinical features explain the observed differences in performance of CRD. An interesting new avenue to improve predictive accuracy of diagnostics for food allergy is to combine CRD with demographic and clinical parameters into predictive models (22, 23). The most important parameter associated with mild symptoms is having a pollen allergy, even if seen together primary sensitization to molecules associated with severe symptoms. The mechanism of such a “protective” effect remains to be elucidated. The overall picture emerging is that skin-associated outcomes (atopic dermatitis ever in life, symptoms upon skin contact with the culprit food, latex allergy) are associated with an increased risk of severe reactions. Whether these observations can be explained by the original route of sensitization being the skin remains to be explored. These studies do demonstrate the potential to improve food allergy diagnosis by using computer algorithms that combine data from different sources. In an era of omics and artificial intelligence it is expected that combinations of old and new biomarkers will further improve diagnostic accuracy.

## Food Allergy: How to Treat It?

AIT for respiratory allergies is an established effective and safe treatment with evidence for sustained tolerance, both for the subcutaneous and the sublingual route. For food allergy, oral (OIT), epicutaneous (EPIT), sublingual (SLIT), and subcutaneous (SCIT) treatments are at different stages of development (24, 25). Until now, the only AIT for food allergy that received market authorization is OIT for peanut. Major challenges for AIT for food allergy are the occurrence of serious side effects, a limited effect size and above all the lack of evidence for sustained efficacy. Serious side-effects are mainly associated with OIT. Although the effect-size for OIT is quite good, sustained efficacy upon termination of the treatment is the exception rather than the rule (26). For EPIT and SLIT, effect sizes are more modest, but the treatments are well-tolerated. Sustained efficacy has not yet been thoroughly investigated. For SCIT, no efficacy data are available yet. The challenge for the future is to develop a treatment that is safe and provides sustained efficacy. From currently available data, a picture is emerging that efficacy is better when treatment is given to children than to adults. Probably at younger age the immune system is still better amenable to being skewed away stably from allergic inflammation toward tolerance. Support for this assumption also comes from the LEAP and LEAP-ON early intervention studies: children around 1 year of age that were already sensitized to peanut did not develop peanut allergy if peanut was introduced early in life (13, 27). Together, these data suggest that very early AIT, when sensitization has not yet or just translated into clinical food allergy, may be the most promising path forward. Which route(s) of administration will prove to be most successful is still an open question.

## Non-IgE Food Allergies: Knowledge Gap to Be Bridged

Genetic background, mechanism, and molecular triggers as well as diagnostic work-up of celiac disease are well-established (28), but prevention and treatment are lagging behind compared to IgE-mediated food allergy. The only treatment option available is avoidance of gluten-containing foods. A very recent analysis of data obtained within the EAT intervention study provides first bits of evidence that early introduction (in the first year of life) of wheat may prevent development of celiac disease (29). Further evidence for a potentially beneficial effect of early introduction and immunotherapeutic treatment options are challenges for the future.

Other non-IgE mediated food allergies, such as eosinophilic esophagitis (EoE) and food-protein-induced enterocolitis (FPIES) are less well-understood. Whether EoE is indeed non-IgE mediated or not is still a matter of debate (30). Some clearly see a role for IgE, some claim IgG_4_ to be involved. Diagnostic strategies to establish which foods are triggering EoE are long and cumbersome avoidance and reintroduction protocols and reliable alternatives are urgently needed. Also, the mechanism of FPIES is still poorly understood, and the triggers at molecular level have not yet been identified (31). To improve the quality of life of patients suffering from these diseases is a major challenge for the research community.

## Summary

**Figure 1:**
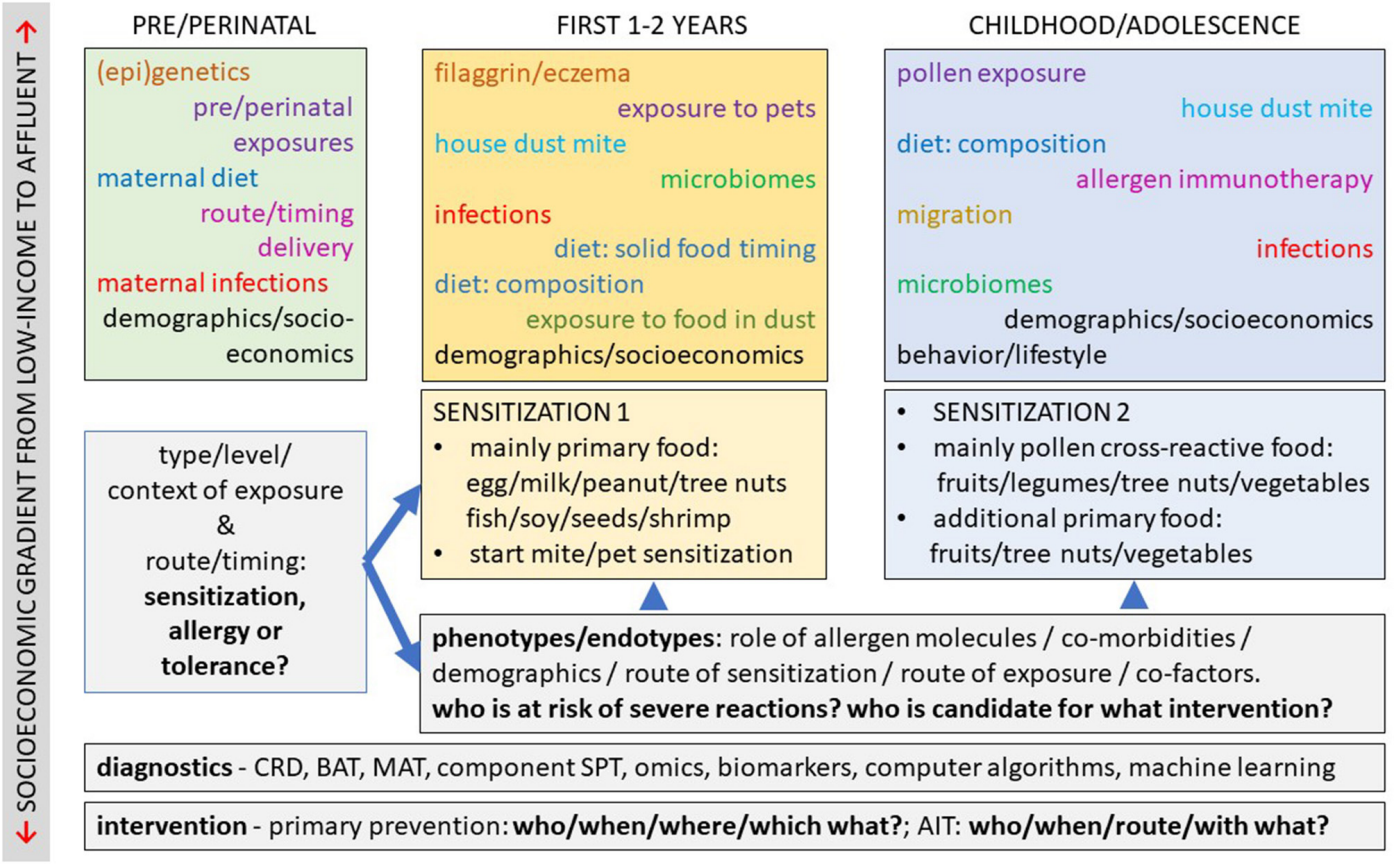
Areas of active research in food allergy. Schematic representation of the current knowledge and major areas of active research in the field of food allergy. In the upper three boxes, endogenous and exogenous factors are listed that are important for the development of food allergy at subsequent stages of life. A combination of those factors, together with route and timing of exposure, decides on tolerance, sensitization or clinical food allergy: from primary food allergy early in life to continued primary, and new cross-reactive food allergies later in childhood and adolescence. Most of these factors differ and evolve along socio-economic gradients across the world, providing a wealth of real-life data. Combination of multi-source data is required for better phenotyping and endo-typing of food allergic patients, using novel biomarkers and diagnostic tests and algorithms. Progress in these fields of translational research will lead to improved prevention, treatment, and management of food allergy.

Food allergy has increased in prevalence and top quality basic and translational research are needed to combat its impact on the quality of life of patients and their caretakers. Better understanding of the mechanisms behind this increase should capitalize on comparative studies in affluent countries, upcoming economies and low-income countries. This will help designing better preventive and therapeutic strategies. Elucidating the routes and mechanisms of sensitization will allow to better distinguish phenotypes and endotypes of food allergy. Progress in these areas of research is essential for development of effective preventive strategies and therapeutic options. Early intervention has gained lots of support, but confirmation is needed in other populations and for other foods. Public health policies will need to be evidence-based, taking local population, culture, and lifestyle into account. Combining *in vitro* serological (CRD) and cellular (BAT) assays with demographic and clinical data into prediction models may prove to be a promising avenue to reduce dependency on DBPCFC for diagnosis, but validation of such approaches is urgently needed. AIT is rapidly gaining ground in food allergy, but major challenges with respect to safety and sustained efficacy are lying ahead. Finally, the origins and mechanisms of non-IgE mediated food allergies need to be further investigated to facilitate preventive and therapeutic strategies. The Food Allergy Section of Frontiers in Allergy welcomes high quality contributions to all these and adjacent fascinating areas of research (Figure 1).

## Author Contributions

The author confirms being the sole contributor of this work and has approved it for publication.

## Conflict of Interest

The author declares the following potential conflicts of interest: Consultancies for HAL Allergy BV, Citeq BV, and Angany Inc; Speaker's fees from HAL Allergy BV and ThermoFisher Scientific.
